# Indulging in Smartphones in Times of Stress: A Moderated Mediation Model of Experiential Avoidance and Trait Mindfulness

**DOI:** 10.3390/bs12120485

**Published:** 2022-11-29

**Authors:** Junjie Zhang, Enna Wang

**Affiliations:** 1 Collaborative Innovation Center of Assessment toward Basic Education Quality, Beijing Normal University, Beijing 100875, China; 2 School of Education, Tianjin University, Tianjin 300350, China

**Keywords:** perceived stress, experiential avoidance, problematic smartphone use, trait mindfulness, college students

## Abstract

Although previous studies have shown that perceived stress is positively related to problematic smartphone use, knowledge of mediating and moderating mechanisms underpinning this relationship is quite limited. In this study, we explored whether experiential avoidance mediated the relationship between perceived stress and problematic smartphone use and whether trait mindfulness moderated this mediating process. A total of 763 Chinese college students completed the measures of perceived stress, experiential avoidance, problematic smartphone use, and trait mindfulness. The results indicate that perceived stress was positively related to problematic smartphone use and this relation was partially mediated by experiential avoidance. Furthermore, moderated mediation analysis showed that trait mindfulness moderated the linkage between perceived stress and problematic smartphone use via experiential avoidance. This link became weaker for college students with higher levels of trait mindfulness. The results highlight the value of identifying the underlying mechanisms between perceived stress and college students’ problematic smartphone use.

## 1. Introduction

With the advancement of technology, especially the popularity of smartphones, people around the world can engage in a vast array of online activities. There are as many as 989 million Internet users in China, and 99.7% of them use phones to access the Internet [1]. The spread of mobile phones has made interpersonal communication more convenient and exerted a positive influence on college students’ everyday living, such as maintaining social connections [2] and increasing course satisfaction [3]. However, college students who use smartphones often fall into problematic situations, such as digital addiction [4], problematic cybersex behavior [5], and cyberbullying perpetration [6]. In particular, the prevalence of problematic smartphone use is high among undergraduates, with 14.5–46% having addictive smartphone use both in China [7,8] and in Western countries [9], and this rate is still rapidly climbing.

Defined as “an individual’s out-of-control behavior due to the excessive usage of mobile phones”, problematic smartphone use (also referred to as smartphone addiction) is considered as a type of behavioral addiction, since it caters to the criteria of craving (obsessive thoughts and feelings about mobile phones), tolerance (an increasing amount of time spent on mobile phones), and withdrawal (durative anxiety when without mobile phones) [10,11]. Emerging research has suggested that problematic smartphone use, smartphone addiction, or excessive smartphone use may directly lead to a series of functional impairments and distress in undergraduates [12,13,14]. Those who are addicted to their smartphones usually experience various harmful consequences ranging from physical symptoms, such as poor sleep quality [15] or psychosomatic symptoms, such as depression and anxiety [16,17], to social problems, such as worse face-to-face interpersonal interaction and even social–emotional distress [18]. Considering these possible adverse consequences, exploring the factors that may bring about an increase in college students’ problematic smartphone use is of immense theoretical implication and practical value.

### 1.1. Perceived Stress and Problematic Smartphone Use

Perceived stress refers to threatening or otherwise demanding situations where individuals have no sufficient ability or available resources to cope with the stress [19]. It usually arises when an individual subjectively appraises a situation or an event as threatening to the achievement of important targets or overwhelming to their resources. Individuals who felt stressed were more likely to engage in addictive behaviors (e.g., Internet addiction and short-form video application addiction) [20,21,22,23]. This effect can usually be illustrated by the general tension theory [24,25], which attributes problematic behaviors, including addictive behaviors, mainly to negative emotions and affects resulting from a variety of stresses, such as failing to accomplish important goals, being unable to maintain valued stimuli, or experiencing noxious events. Built off this theory, uncomfortable emotions arising in reaction to perceived stress increase the likelihood of being addicted to one’s smartphone. Previous studies also suggest that perceived stress is one of the antecedents of problematic smartphone use in both adolescent samples and university student samples [26,27]. For instance, a study found that adolescents who reported high perceived stress were most likely to become involved in smartphone addiction [26]. In another study, researchers using a sample of 628 college students also emphasized the significance of increasing perceived stress in reinforcing smartphone addiction during the COVID-19 epidemic [27]. These findings provide evidence to confirm that perceived stress is a risky factor underlying the appearance of problematic smartphone use; however, the existing literature fails to uncover the possible mechanisms between perceived stress and problematic smartphone use. Thus, we attempted to reveal how perceived stress contributes to smartphone addiction and what buffering factors might minimize their connection and may be pivotal for shedding insight on developing effective and targeted smartphone addiction intervention programs.

### 1.2. Experiential Avoidance as a Mediator

Viewed through the lens of acceptance and commitment therapy, many problematic behaviors, despite their apparent dissimilarity, could use the same and common explanatory mechanism of experiential avoidance [28,29]. Experiential avoidance refers to the strategy that individuals use to control or escape from uncomfortable stimuli (e.g., negative thoughts and feelings) [30]. Unlike the general concept of avoidance, experiential avoidance emphasizes the individual’s avoidance of inner experiences, rather than outer objective things [31]. As a short-term, self-regulation strategy, experiential avoidance may facilitate individuals to handle troubling somatic feelings, thoughts, and emotions related to certain negative circumstances [32]. However, in the long run, when it is generalized to other similar negative circumstances by individuals falling into an inflexible model (i.e., experiential avoidance), it may cause greater damage, increase the sense of lack of control, and hinder the acquisition of valuable goals [31,33]. Growing evidence supporting the contributing role of experiential avoidance in the domain of addictions has been found [34,35]. For example, substantial studies adopting a cross-sectional design have indicated that experiential avoidance is positively related to a wide variety of addictive problems, such as drug addiction, alcohol abuse, and food addictions [35,36,37].

For technological addictions, experiential avoidance can also serve as a crucial predictor of Internet addiction as well as video game addiction [38]. Some studies have also confirmed that there is a closed linkage between experiential avoidance and problematic smartphone use [39,40]. One potential interpretation for this relationship may be that considering the accessibility, convenience, and privacy of smartphones, smartphone use as a “safety behavior” [41] could serve as the most common way to apply experiential avoidance to relieve unpleasant experiences or emotional distress triggered by aversive stimuli (e.g., perceived stress) [38,42]. Thus, individuals slowly develop an addiction to smartphones over time. Previous studies have also revealed that higher perceived stress usually leads to a lack of psychological flexibility [43,44,45]. One recent study showed that perceived stress has a significant negative relationship with experiential avoidance [46]. That is, individuals high in perceived stress are prone to avoid unpleasant emotions or uncomfortable experiences, and they are more likely to adopt experiential avoidance, thereby escaping unwanted feelings.

According to the theory of acceptance and commitment therapy [30], experiential avoidance, which serves as a function to control or avoid unwanted events, is a common explanatory mechanism between stressful internal stimuli (e.g., perceived stress) and many problematic behaviors (e.g., drug abuse and gambling). Cumulative evidence has supported Hayes, Wilson, Gifford, Follette, and Strosahl’s [30] proposition that experiential avoidance mechanisms can make individuals escape from stressful internal stimuli, and once the inflexible experiential avoidance pattern is confirmed, maladaptive behaviors are more likely to occur. For example, recent studies have suggested that experiential avoidance can mediate the relationship between unwanted stimuli and addictive behaviors, such as perceived stress and tobacco dependence, as well as shame and Internet addiction [47,48]. More specifically, one study using 307 Chinese undergraduate students found that experiential avoidance can fully mediate the relationship between shame and Internet addiction [47]. It should be noted that compared with computers, due to the convenience and accessibility of smartphones, undergraduates could use their smartphones at anytime and anywhere. As such, it is usually taken as an easily accessible way to escape stressful events or uncomfortable experiences for undergraduates. Therefore, it is reasonable to speculate that college students who experience high stress are more likely to adopt experiential avoidance and consequently become indulged in smartphones. Thus, we proposed the following hypothesis:

**Hypothesis** **1.** *Experiential avoidance would mediate the relationship between perceived stress and problematic smartphone use.*

### 1.3. Trait Mindfulness as a Moderator

Although perceived stress may affect problematic smartphone use through the mediating effect of experiential avoidance, not all students under stress display higher levels of experiential avoidance and experience a high level of excessive smartphone use. Therefore, it is crucial to tap into those factors that can be utilized to buffer or weaken the effect of perceived stress on smartphone addiction as well as experiential avoidance on smartphone addiction. In the current study, we hypothesized that trait mindfulness moderated the relationship between perceived stress and problematic smartphone use as well as perceived stress and experiential avoidance. Trait mindfulness refers to the ability to be aware of the present moment and one’s experiences with an attitude of acceptance and nonjudgment [49,50] and is assumed to be cultivated or changed through regular practice (e.g., MBSR training) [51]. An extant study found that mindfulness is positively associated with subjective well-being and negatively linked to problematic behaviors [52]. Moreover, accumulated evidence has shown that individuals with higher trait mindfulness are less likely to exhibit smartphone addiction behavior [53,54,55], and prior studies have also revealed that increased mindfulness can effectively alleviate individual abuse of mobile phones through mindfulness-based training courses [56,57].

The reperceiving model of mindfulness posits that mindfulness could help people to reperceive the inner and external experience objectively, break free from automatic response patterns, and develop adaptive reactions to negative stimuli [58]. More specifically, mindfulness helps individuals reperceive stressful experiences with an objective and nonjudgmental attitude, stop automated avoidance responses, such as experiential avoidance, and develop more appropriate coping instead of dwelling on the smartphone. In light of this model, individuals with high traits of mindfulness are capable of paying attention and being aware of the present without judging it. This enables them to objectively reperceive what they are thinking and feeling, especially when they are under pressure and thus prevent experiential avoidance and problematic smartphone use from happening [58,59]. As a result, college students who possess higher levels of trait mindfulness appear to be less likely to experience experiential avoidance and problematic smartphone use when faced with stress.

Furthermore, the risk-buffering model also assumes that certain protective buffers can attenuate the undesirable effects of risk factors [60]. In other words, as a positive protective factor, mindfulness can buffer the negative effects of various risk factors on individuals. Previous empirical studies support this hypothesis. For instance, Liu et al. [61] found that trait mindfulness can moderate the linkage between an adverse family environment and addiction behaviors such that the positive relationship between family dysfunction and phone addiction is weaker for adolescents with higher levels of trait mindfulness. Similarly, trait mindfulness could reduce the influence of life stressors on both internalizing and externalizing problems; that is, these predictive effects were buffered for adolescents or young adults with high mindfulness [62,63]. Moreover, trait mindfulness could moderate both the direct and indirect effects of perceived press on phone addiction via self-control [26]. On the basis of the risk-buffering mode, the detrimental effect of perceived stress on maladaptive response will be dampened under the effect of high trait mindfulness. To our knowledge, however, no prior study has verified whether trait mindfulness is a buffer that attenuates the negative influence of perceived stress on college students’ experiential avoidance and problematic smartphone use. Thus, we proposed the following hypothesis:

**Hypothesis** **2.** *Trait mindfulness would moderate the direct or indirect relationship between perceived stress and problematic smartphone use via experiential avoidance. To be more specific, the linkage between perceived stress and experiential avoidance as well as perceived stress and problematic smartphone use would be much weaker for college students with higher levels of trait mindfulness.*

### 1.4. The Present Study

**Figure 1:**
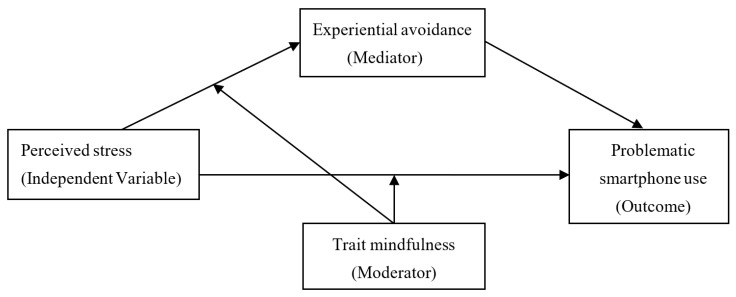
The proposed moderated mediation model.

In a nutshell, the present study aims to address two questions: How does perceived stress lead to problematic smartphone use and when is the linkage the most potent? Based on the theory of acceptance and commitment therapy, reperceiving model of mindfulness and risk-buffering model, the two questions above constitute a moderated mediation model, in which we could examine whether experiential avoidance would mediate the association between perceived stress and problematic smartphone use (the mediation mechanism), as well as whether the direct or indirect association between perceived stress and problematic smartphone use through experiential avoidance would be moderated by trait mindfulness (the moderation mechanism), see Figure 1 for details.

## 2. Method

### 2.1. Participants and Procedure

The present study was conducted in 2021 at four universities in Guangdong Province, China. We collected initial data on a frequently used online survey platform in China, the Questionnaire Star Survey [64,65] and distributed questionnaires to participants via university counselors. Before this survey started, the purposes and procedures of our study were explained in a vague way (e.g., to explore what factors may influence smartphone use) to reduce response bias. All participants were informed that the survey was anonymous and would be treated in confidence, and they were free to discontinue their participation at any time.

A total of 800 Chinese undergraduates signed informed consent forms and completed the questionnaires. After data cleansing procedures, 37 unqualified samples (4.63%) were excluded for answering regularly, completing the survey with too short a duration, and missing all three attention checks (e.g., Please choose “agree” for this item). The final sample consisted of 763 participants, 291 males and 472 females. Their ages ranged from 17 to 23 (*M_age_* = 20.28, *SD_age_* = 1.56). The research was conducted under the Declaration of Helsinki, and the protocol was approved by the Ethics Committee of the first author’s affiliation institution.

### 2.2. Measures

#### 2.2.1. Perceived Stress

Perceived stress was measured using the 10-item Chinese version of the Perceived Stress Scale Short Form (PSS-10), which assesses the degree of subjective stress in a person’s life and how they perceive their ability to cope with stressful situations [66,67]. Among the 10 items, 4 items were positively worded; a representative item was: “How often did you feel confident about your ability to handle your personal problems?”, and the remaining 6 items were negatively worded; a representative item was: “How often did you feel difficulties were piling up so high that you could not overcome them?” Individuals rated these items on a 5-point Likert response format ranging from 0 (never) to 4 (very often). After reversely coding the 4 positive items, we computed the average score of 10 items. A higher score represented greater perceived stress. For the present study, the Cronbach’s α of this scale was 0.86.

#### 2.2.2. Problematic Smartphone Use

Problematic smartphone use was assessed by the Chinese Short Version of the Smartphone Addiction Scale (SAS-SV) [68,69]. Participants were asked to rate the 10 items on a 6-point Likert response format (1 = strongly disagree to 6 = strongly agree). A representative sample was: “Having my smartphone in my mind even when I am not using it.” The scores of the 10 items were averaged, with higher scores denoting greater problematic smartphone use. In this study, Cronbach’s α for the SAS-SV was 0.88.

#### 2.2.3. Experiential Avoidance

The Acceptance and Action Questionnaire-II (AAQ-II) was initially developed by Bond et al. [70], and the Chinese edition was revised by Cao et al. [71] and used to assess experiential avoidance in this study. Participants respond to 7 items on a 1 (strongly disagree) to 7 (strongly agree) scale. A representative sample was: “I worry about not being able to control my worries and feelings.” The mean score of 7 items was computed in this study, with higher scores representing a high level of avoidance when experiencing uncomfortable sensations or thoughts. For the current study, Cronbach’s α for the AAQ was 0.88.

#### 2.2.4. Trait Mindfulness

The Chinese version of the Five Facet Mindfulness Questionnaire Short Form (FFMQ-C-SF) developed by Zhu et al. [72] was used to measure the participants’ levels of trait mindfulness. This short-form scale consisted of 15 items, with 3 items assessing each of the five domains including observing, describing, acting with awareness, nonjudging of inner experience, and nonreactivity to inner experience. A representative item was: “I remain present with sensations and feelings even when they are unpleasant or painful.” Respondents were required to rate items using a 5-point Likert response format ranging from 1 (almost never) to 5 (almost always). The mean value of the total scores across the 15 items was computed, with higher scores on behalf of higher levels of trait mindfulness. In the current study, the Cronbach’s α of this scale was 0.82, and the Cronbach’s alphas of five subscales ranged from 0.72 to 0.86.

### 2.3. Data Analysis

To test Hypothesis 1 and Hypothesis 2 of the present study, descriptive statistics and correlation analysis for these interest variables were calculated using SPSS Statistics 24.0. Next, following the four-step procedure of MacKinnon [73], we examined the potential mediating role of experiential avoidance on the association between perceived stress and problematic smartphone use. This procedure required (a) a significant predictive effect of perceived stress on problematic smartphone use; (b) a significant predictive effect of perceived stress on experiential avoidance; (c) a significant predictive effect of experiential avoidance on problematic smartphone use while controlling for perceived stress; and (d) a significant indirect path between perceived stress and problematic smartphone use via experiential avoidance. After performing the first three steps, we employed the bias-corrected percentile bootstrap method to determine whether the last condition was qualified. Finally, we conducted the analysis of the moderated mediation model using Hayes’s PROCESS macro (Model 8) [74] to examine whether trait mindfulness moderated the mediation process. Notably, before conducting the examination of the mediation model and the moderated mediation model, the data of perceived stress, experiential avoidance, problematic smartphone use, and trait mindfulness were all standardized.

## 3. Results

### 3.1. Testing of Common Method Biases

Because of the self-report method of data collection from a single source, we additionally tested for the common method bias using Harman’s one-factor test. This test showed that the variance interpretation rate of the first common factor was 30.14%, less than the critical standard of 40% [75], indicating that the influence of the common method bias in this study was not of great concern.

### 3.2. Preliminary Analyses

Table 1 shows the means, standard deviations, and correlations for the key variables utilized in each analysis. As expected, both perceived stress and experiential avoidance were positively correlated with problematic smartphone use. In contrast, trait mindfulness was negatively correlated with smartphone addiction. In addition, perceived stress was positively correlated with experiential avoidance, whereas it was negatively correlated with trait mindfulness. Experiential avoidance was negatively correlated with trait mindfulness. In addition, male students, compared to female students, reported significantly less perceived stress, experiential avoidance, and problematic smartphone use.

### 3.3. Testing for Mediation Effect

In Hypothesis 1, we anticipated that experiential avoidance would mediate the relationship between perceived stress and problematic smartphone use. Following MacKinnon’s 4-step procedure [73], we examined the mediation effect of experiential avoidance. In all analyses, we controlled for gender as a covariate. The multiple regression analysis showed that perceived stress was significantly associated with problematic smartphone use, *β* = 0.40, *p* < 0.001 (the first step, see Model 1 of Table 2). Perceived stress was significantly associated with experiential avoidance, *β* = 0.66, *p* < 0.001 (the second step, see Model 2 of Table 2). After controlling for perceived stress, experiential avoidance was significantly associated with problematic smartphone use, *β* = 0.23, *p* < 0.001 (the third step, see Model 3 of Table 2). In the final step, we used the bias-corrected percentile bootstrap method to examine the mediating effect of experiential avoidance. Our results indicate that (a) the total effect of perceived stress on problematic smartphone use was significant, total effect = 0.40, SE = 0.03, *t* = 12.15, *p* < 0.001, 95% CI = [0.34, 0.47]; (b) the indirect effect of perceived stress on problematic smartphone use via experiential avoidance was significant, indirect effect = 0.15, Boot SE = 0.03, BootLLCI = 0.09, BootULCI = 0.22; (c) the direct effect of perceived stress on problematic smartphone use was significant, direct effect = 0.25, SE = 0.04, *t* = 5.71, *p* < 0.001, 95% CI = [0.16, 0.33]. The mediation effect accounted for 38.27% of the total effect. Thus, our results fully meet the four requirements for constructing the mediation effect, which suggested that Hypothesis 1 was supported.

### 3.4. Testing for Moderated Mediation

In Hypothesis 2, we assumed that trait mindfulness would moderate the direct/indirect relations between perceived stress and problematic smartphone use via experiential avoidance. Using the PROCESS macro process (Model 8), we examined this moderated mediation model. Specifically, we estimated parameters for two regression models. The relationship between perceived stress and experiential avoidance as well as the moderating effect of trait mindfulness on this relationship were examined in Model 1. The moderating effect of trait mindfulness on the relationship between perceived stress and problematic smartphone use was estimated in Model 2. In these two models, gender was controlled as a covariate (see Table 3 for details).

**Figure 2:**
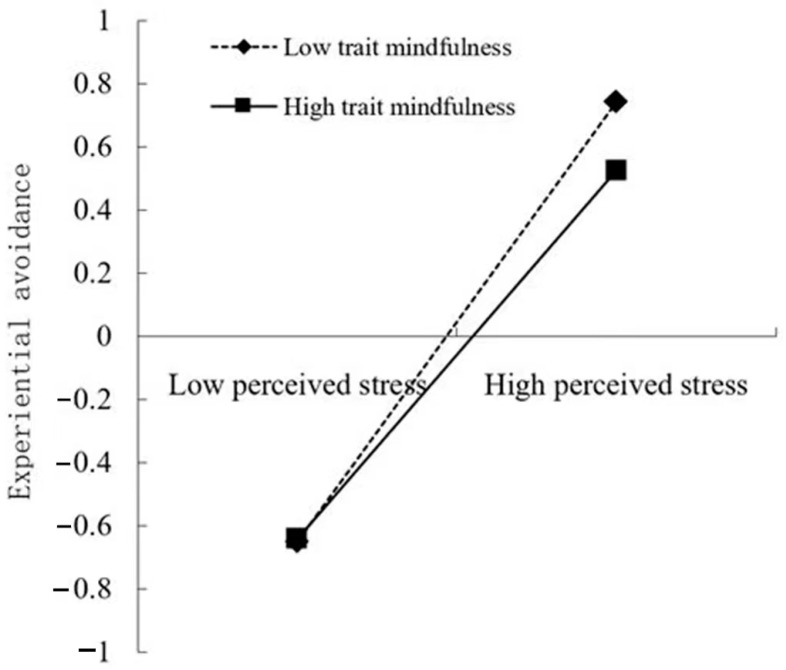
Plot of the association between perceived stress and experiential avoidance at two levels of trait mindfulness.

As Table 3 illustrates, Model 1 showed that the effect of perceived stress on experiential avoidance was significant, *β* = 0.64, *p* < 0.001 and buffered by trait mindfulness, *β* = 0.06, *p* < 0.01. Model 2 showed that the effect of perceived stress on problematic smartphone use was significant, *β* = 0.22, *p* < 0.001, but not moderated by trait mindfulness, *β* = −0.004, *p* < 0.05. For an intuitive description, this study plotted perceived stress on experiential avoidance separately for low levels of trait mindfulness (1 SD below the mean) and high levels of trait mindfulness (1 SD above the mean), see Figure 2. Simple slope tests illustrated that for college students with low trait mindfulness, perceived stress was significantly associated with experiential avoidance, *β_simple_* = 0.70, *p* < 0.001. For college students with high trait mindfulness, this association was still significant but relatively weaker, *β_simple_* = 0.58, *p* < 0.001.

Furthermore, we used the bias-corrected percentile bootstrap method to verify the moderated mediation model. The results indicate that the indirect effect of perceived stress on problematic smartphone use via experiential avoidance was buffered by trait mindfulness. For college students with low levels of trait mindfulness, the indirect effect of perceived stress on problematic smartphone use via experiential avoidance was significant, ab = 0.16, SE = 0.03, 95% CI = [0.09, 0.23]. For college students with high levels of trait mindfulness, perceived stress had a weaker effect on problematic smartphone use through experiential avoidance, ab = 0.13, SE = 0.03, 95% CI = [0.07, 0.19]. Thus, the results partially supported Hypothesis 2.

## 4. Discussion

A recent meta-analysis showed that the overall global prevalence of problematic smartphone use is 28.3%, much higher than other types of behavioral addictions [76], and problematic smartphone use may become one of the most important behavioral addictions in the 21st century [77]. Considering the high prevalence of problematic smartphone use and its various negative effects, this study focused on the relationship and mechanisms between perceived stress and problematic smartphone use among Chinese college students. The effect of stress on problematic smartphone use has gained ample empirical support (e.g., [26,27,78,79]). Unfortunately, questions regarding the mechanisms that mediate and moderate the relationship between perceived stress and problematic smartphone use remain largely unanswered. In this study, our findings indicate that the unfavorable effect of perceived stress on problematic smartphone use was partially explained by increased experiential avoidance. Furthermore, the indirect relation between perceived stress and problematic smartphone use was moderated by trait mindfulness such that the path from perceived stress to experiential avoidance (the first stage of the mediation process) was weakened in the context of higher trait mindfulness. In the following section, we will discuss in detail our two hypotheses regarding this moderated mediation model of perceived stress and problematic smartphone use.

### 4.1. The Mediating Role of Experiential Avoidance

In line with Hypothesis 1, our results show that experiential avoidance partially explained the adverse effect of perceived stress on problematic smartphone use. Although earlier studies have revealed that experiential avoidance is an essential mediation mechanism bridging stressful, unpleasant, or unwanted internal stimuli (e.g., perceived stress and shame) with maladaptive behaviors (e.g., tobacco dependence and Internet addiction) [47,48], this study is the first study to demonstrate the mediating role of experiential avoidance in the linkage between perceived stress and problematic smartphone use. These findings transcend the previous literature on why perceived stress may activate young adults’ problematic smartphone use through self-control, negative emotion, or rumination [26,27]. Unlike studies that ignored the potential association between experiential avoidance and smartphone addiction [38], we innovatively took it into consideration and tested the mediating role of experiential avoidance in this linkage. This finding is in accord with the theory of acceptance and commitment therapy [30,80]. Namely, as a construct of acceptance and commitment therapy, experiential avoidance refers to the self-regulation strategy individuals use to control or escape from these stressful internal feelings and thus can work as an explanatory mechanism to build a bridge between perceived stress and addictive behaviors.

Apart from the whole mediation results, it is worth noting the two separate links in our mediation model. For the path from perceived press to experiential avoidance (the first stage of the mediation process), our results support the premise that perceived stress can facilitate the activation of experiential avoidance mechanisms. This finding accords with previous studies [44,81]. That is, individuals who experience high levels of perceived stress have to experience negative emotions or terrible moods, which may create conditions for individuals to adopt experience avoidance. This result could be attributed to the deficit in mental resources young adults use to cope with stressful situations. To be concrete, stress can deplete coping resources, leading individuals to use inappropriate coping strategies and reduced flexibility in dealing with stress. Additionally, individuals with high levels of stress usually have defects in psychological flexibility in resolving uncomfortable internal feelings [43,44]. For the path from experiential avoidance to problematic smartphone use (the second stage of the mediation model), our results indicate that experiential avoidance would result in problematic smartphone use, which is congruent with the theory of acceptance and commitment therapy [82] and the previous empirical study revealing that individuals with higher levels of experiential avoidance have a greater probability of engaging in addictive behaviors [38]. That is, smartphones could offer a way for experiential avoidance so as to make unpleasant experiences or emotional distress appear less harmful to themselves [41,42].

### 4.2. The Moderating Role of Trait Mindfulness

The second objective of this study was to verify the moderating effect of trait mindfulness on the direct or indirect relationship between perceived stress and problematic smartphone use via experiential avoidance. Our findings indicate that the indirect relationship between perceived stress and problematic smartphone use via experiential avoidance was moderated by trait mindfulness. However, trait mindfulness only moderated the first stage of the mediation process (i.e., the relationship between perceived stress and experiential avoidance). These results can be explained by the reperceiving model of mindfulness [58]. On the basis of this viewpoint, individuals high in trait mindfulness can be open to the present experience with nonjudgmental attention and awareness and reperceive their own feelings and thoughts more objectively, accept their inner experience in spite of whether they were positive or negative, and thus block the occurrence of experiential avoidance [58,59]. Thus, for college students with higher levels of trait mindfulness, the relationship between perceived stress and experiential avoidance becomes much weaker. However, contrary to our hypothesis, trait mindfulness does not relieve the direct relationship between perceived stress and problematic smartphone use. This is not in line with the prior findings that the linkage between objective or subjective stress and nonadaptive behaviors is moderated by trait mindfulness [26,62,63]. A possible explanation for this finding may be that college students who perceive high subjective stress have inflexible behavioral response patterns and are inclined to act automatically (e.g., uncontrolled smartphone use) upon feelings of tension and discomfort rather than to experience their feelings with nonreactivity [83]. Despite this, it would be premature to dismiss the importance of trait mindfulness, because more research should be conducted before drawing any definitive conclusions about how trait mindfulness alters the effect of perceived stress on problematic smartphone use.

By taking into account trait mindfulness as a boundary condition in the mediating model, this study identified in a nuanced way the moderating effects that might have been skipped, offering stronger predictive power than a simple mediation model. In the present study, our results indicate that the indirect effect of perceived stress on problematic smartphone use via experiential avoidance becomes weaker for college students with high trait mindfulness, than for those with low trait mindfulness. What we found has a very special significance for Chinese undergraduates. Chinese college students have multiple sources of stress and perceive high levels of stress [84,85], which may lead them to avoid unpleasant experiences and indulge in the world of smartphones to escape from inner strain. As a consequence, perceived stress may have particularly pernicious effects on college students’ problematic smartphone use during their college years. From this, it appears that researchers with an interest in the adverse effect of perceived stress on smartphone addiction should work on fostering trait mindfulness.

## 5. Strength and Limitation

Our findings throw light on both the theoretical research about the internal mechanism of perceived stress in problematic smartphone use and the practical work on the intervention of smartphone addiction. As a theoretical contribution, our study adds to a growing body of literature linking perceived stress and problematic smartphone use, confirming that university students with high perceived stress are more likely to be addicted to smartphones than others with low perceived stress, which is in accordance with previous studies [26,78,79]. Furthermore, it expands our understanding of how perceived stress relates to problematic smartphone use by revealing the mechanisms mediating and moderating the relationship between them, which remains mostly unanswered so far. Concretely speaking, our study appears to be the first study to show that experiential avoidance mediates the link between perceived stress and problematic smartphone use from the theory of acceptance and commitment therapy, which provides a new understanding of why perceived stress may activate individuals’ problematic smartphone use and deserves further study in the future. Moreover, this study extends previous studies by uncovering the moderating role of trait mindfulness between perceived stress and experiential avoidance. That is, the relationship between perceived stress and problematic smartphone use through experiential avoidance is weaker for college students with high levels of trait mindfulness. By incorporating trait mindfulness into the mediating model, moderating effects that might have been skipped were identified in a nuanced way in this study, providing greater predictive power than simple mediation models.

From a practical perspective, our findings will help to reduce the rate of problematic smartphone use among undergraduates and contribute to orienting or planning effective psychological interventions to prevent smartphone addiction. First, by alleviating the perceived stress of college students, we have the potential to reduce their likelihood of problematic smartphone use, yet further studies will be needed to clarify what the possible stressors are among college students and how to alleviate them. Second, we should apply various strategies from the area of acceptance and commitment therapy, such as acceptance-based behavioral interventions to enhance college students’ psychological flexibility and decrease their experiential avoidance [80,82], thus ultimately reducing their problematic smartphone use. In addition, we could also improve the trait mindfulness of college students to relieve the adverse effect of perceived stress on problematic smartphone use via experiential avoidance. All of the above may help practitioners understand pathways between perceived stress and problematic smartphone use and facilitate the development of effective smartphone addiction prevention programs.

Considering the several limitations below, the results of this study may be interpreted with caution. Firstly, although on the basis of well-established theories and empirical findings, a cross-sectional approach may reveal causal associations [86], and the cross-sectional design of our study may limit the credibility and generalizability of related conclusions. Future research can validate or adjust our moderated mediation model of problematic smartphone use by longitudinal studies or experimental methods. Secondly, all data collected in the current study relied on self-reported questionnaires, which were subjective and affected by the social desirability effect and may have influenced the accuracy or reliability of participants’ responses. Future research should consider acquiring more objective phone usage data to evaluate smartphone dependency via the corresponding application (e.g., [17]). Thirdly, we collected only subjectively perceived stress, not objective stressors, and no information on college students in different study areas. Future research could collect objective stressors from college students and explore the impact of diverse stressors on problematic smartphone use among students in various fields of study. Finally, this study was performed with Chinese college students, which may introduce the question of whether these conclusions could generalize to adolescents or older adults, especially those from other backgrounds. More studies adopting various sample populations are needed to explore the age or cultural oneness of these findings.

## Figures and Tables

**Table 1:** Descriptive statistics and correlations among variables.

Variable	*M*	*SD*	1	2	3	4	5
1. Gender	-	-	1				
2. Perceived stress	2.77	0.59	−0.10 **	1			
3. Experiential avoidance	3.29	1.17	−0.09 *	0.67 ***	1		
4. Trait mindfulness	3.08	0.34	−0.02	−0.40 ***	−0.31 ***	1	
5. Problematic smartphone use	3.31	1.10	−0.15 ***	0.41 ***	0.41 ***	−0.23 ***	1

Note. Gender was dummy coded (1 = male and 0 = female). * *p* < 0.05; ** *p* < 0.01; *** *p* < 0. 001. *n* = 63.

**Table 2:** Testing the mediation effect of perceived stress on problematic smartphone use.

Predictors	Model 1 (Problematic Smartphone Use)					Model 2 (Experiential Avoidance)			Model 3 (Problematic Smartphone Use)			
	*β*	*SE*	*t*	*95% CI*	*β*	*SE*	*t*	*95% CI*	*β*	*SE*	*t*	*95% CI*
Gender	−0.24	0.07	−3.49 ***	[−0.37, −0.10]	−0.06	0.06	−1.03	[−0.17, 0.05]	−0.22	0.67	−3.35 **	[−0.35, −0.09]
Perceived stress	0.40	0.03	12.15 ***	[0.34,0.47]	0.66	0.03	24.43 ***	[0.61, 0.72]	0.25	0.04	5.71 ***	[0.16, 0.33]
Experiential avoidance									0.23	0.04	5.34 ***	[0.14, 0.32]
**R^2^**	0.18						0.44		0.21			
**F**	84.74 ***						304.07 ***		68.06 ***			

Note. The *β* values are standardized regression coefficients. Gender was dummy coded (1 = male and 0 = female). ** *p* < 0.01; *** *p* < 0.001.

**Table 3:** Testing the moderated mediation effect of perceived stress on problematic smartphone use.

Predictors	Model 1 (Experiential Avoidance)				Model 2 (Problematic Smartphone Use)			
	*β*	*SE*	*t*	*95% CI*	*β*	*SE*	*t*	*95% CI*
Gender	−0.05	0.06	−0.82	[−0.16, 0.06]	−0.23	0.07	−3.46 ***	[−0.36, −0.10]
Perceived stress	0.64	0.03	21.66 ***	[0.58, 0.70]	0.22	0.05	4.90 ***	[0.13, 0.31]
Trait mindfulness	−0.05	0.03	−1.75	[−0.11, 0.01]	−0.08	0.04	−2.21 *	[−0.15, −0.01]
Trait mindfulness × Perceived stress	−0.06	0.02	−2.76 **	[−0.10, −0.02]	−0.004	0.02	−0.16	[−0.05, 0.04]
Experiential avoidance					0.22	0.04	5.14 ***	[0.14, 0.31]
**R^2^**			0.45				0.22	
**F**			156.72 ***				41.98 ***	

Note. The *β* values are standardized regression coefficients. Gender was dummy coded (1 = male and 0 = female). * *p* < 0.05; ** *p* < 0.01; *** *p* < 0.001.

## Data Availability

The data that support the findings of this study are available from the corresponding author upon reasonable request.
